# Supplementary material for: Insights into Localized Crystallization in the 3D-Cone Solar Evaporator for High-Salinity Desalination
Source: Materials (Basel). 2025 Jun 3;18(11):2610. doi: 10.3390/ma18112610 (PMC12155923; doi:10.3390/ma18112610)
Supplement: Supplementary file 1 [file materials-18-02610-s001.zip › materials-3657187-supplementary.pdf]

List of Supporting Information

Figure S1. Mass change curves of 3D-cone solar evaporators with different weight percentage optimization of carbon powders under 1 sun irradiation.

Figure S2. Mass change curves of 3D-cone solar evaporators with various H/D in darkness.

Figure S3. (a)(b)(c) Evaporation rates of 24.5 wt.% saline water as a function of time for the 3D-cone solar evaporators with the H/D ratio of 0.25, 1 and 1.75 under 1 sun irradiation.

Figure S4. (a)-(g) Time sequence of optical captures displaying the localized crystallization process on the 3D-cone solar evaporators with the H/D ratio of 0.25, 0.5, 1, 1.25, 1.75 and 2 under 1 sun irradiation.

Figure S5. The digital photographs of the 3D-cone solar evaporators with salt precipitation on evaporators with the H/D of 0.25, 0.5, 1, 1.25, 1.75 and 2.

Figure S6. Comparison of solar desalination performance and previous reports under one sun illumination, including the evaporation rate and their salt tolerance in different salt concentration.

Supplementary Table S1. The 3D-cone solar evaporators with salt precipitation on evaporators with the variation of H/D ratios.

Supplementary Table S2. Summary of representative references on solar-driven desalination with the variation of salt concentration.

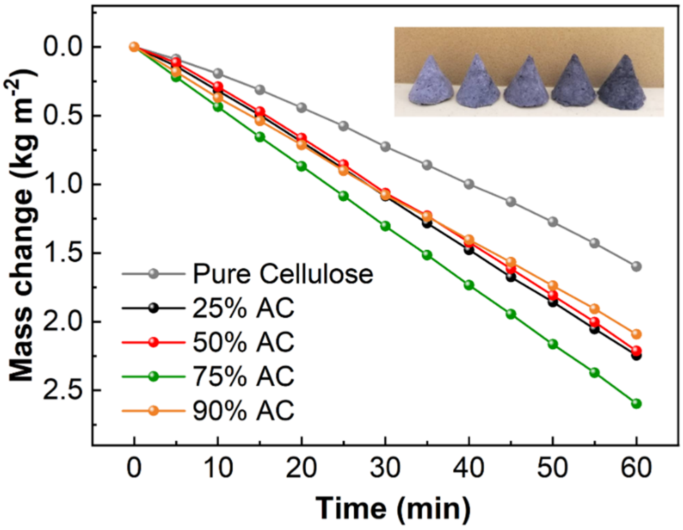

Figure S1. Mass change curves of 3D-cone solar evaporators with different weight percentage optimization of carbon powders under 1 sun irradiation.

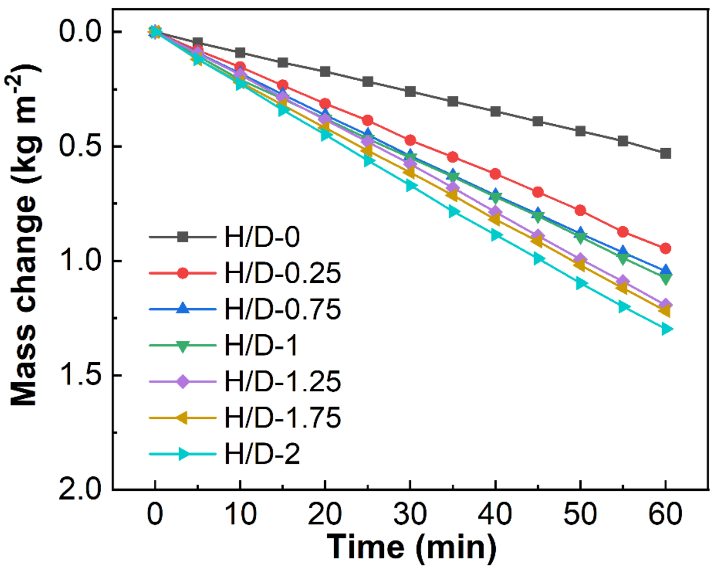

Figure S2. Mass change curves of 3D-cone solar evaporators with various H/D in darkness.

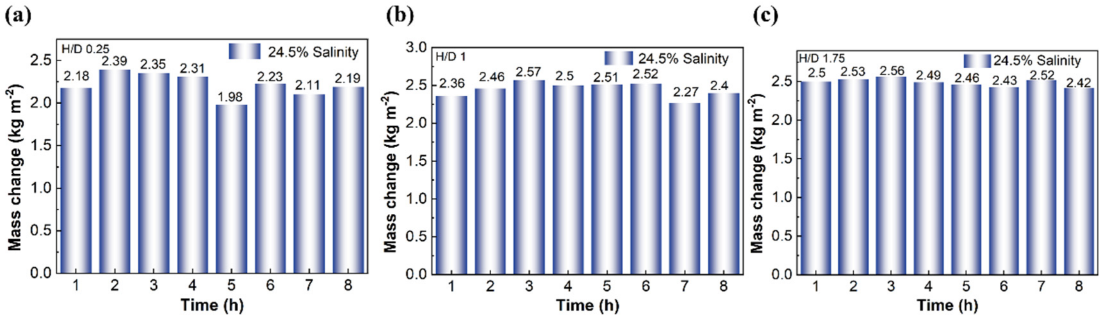

Figure S3. (a)(b)(c) Evaporation rates of 24.5 wt.% saline water as a function of time for the 3D-cone solar evaporators with the H/D ratio of 0.25, 1 and 1.75 under 1 sun irradiation.

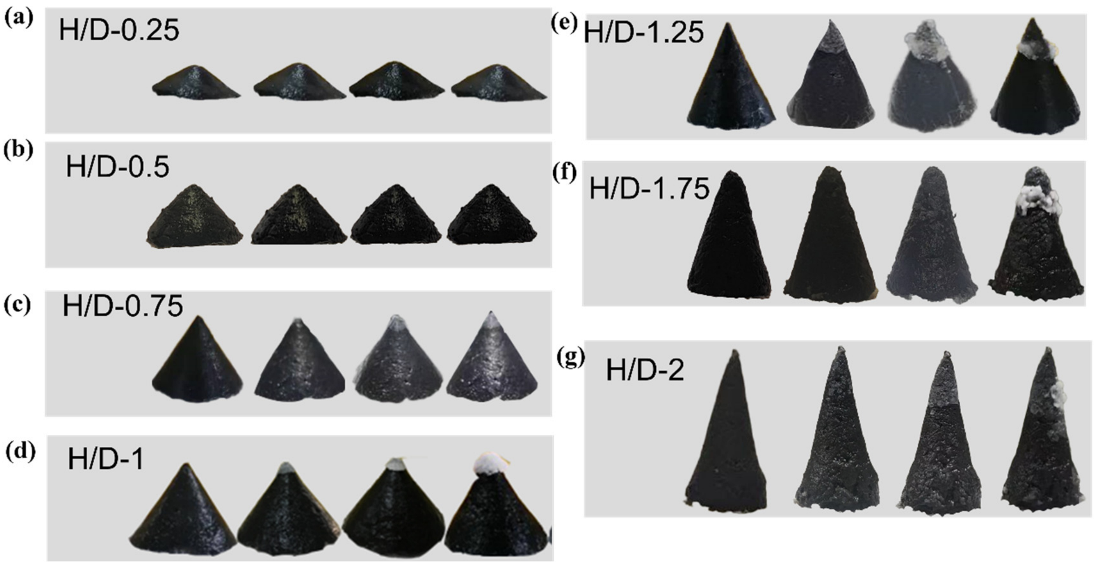

Figure S4. (a)-(g) Time sequence of optical captures displaying the localized crystallization process on the 3D-cone solar evaporators with the H/D ratio of 0.25, 0.5, 1, 1.25, 1.75 and 2 under 1 sun irradiation.

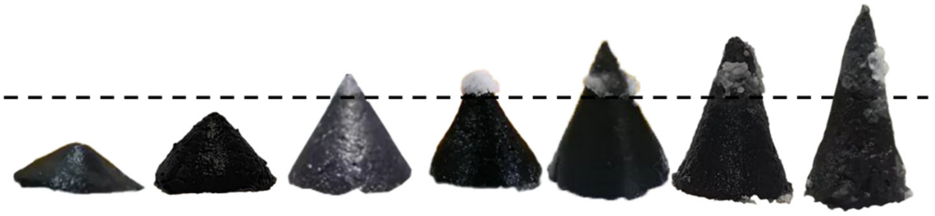

Figure S5. The digital photographs of the 3D-cone solar evaporators with salt precipitation on evaporators with the H/D of 0.25, 0.5, 1, 1.25, 1.75 and 2.

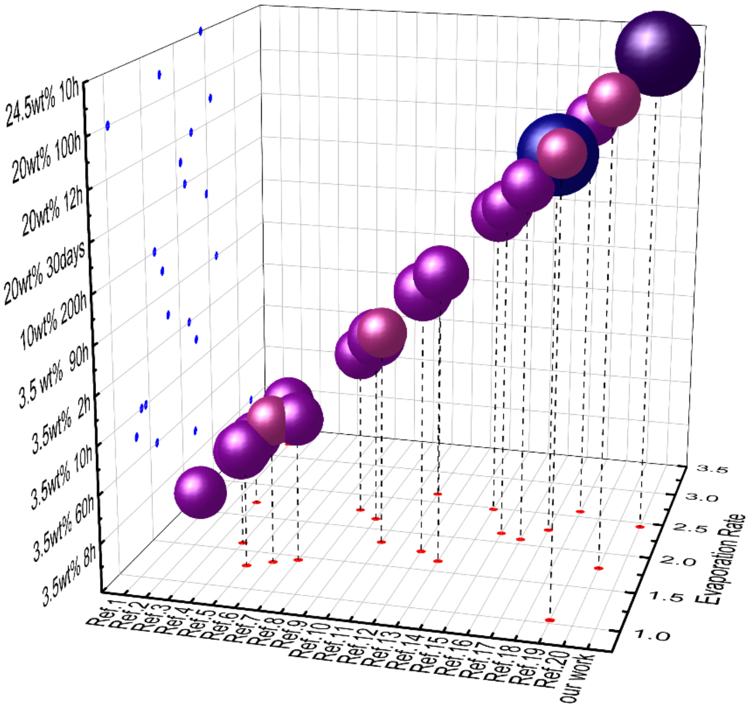

Figure S6. Comparison of solar desalination performance and previous reports under one sun illumination, including the evaporation rate and their salt tolerance in different salt concentration [1-20].

Supplementary Table S1 The 3D-cone solar evaporators with salt precipitation on evaporators with the variation of H/D ratios.

**H/D**	**Out of salt time**	**Out of salt distance**
0.25	No salt	0 mm
0.5	No salt	0 mm
0.75	60 min	~1 mm
1	70 min	~7 mm
1.25	100 min	~10 mm (salt dissolve)
1.75	120 min	~16 mm (salt dissolve)
2	150 min	~22 mm (salt dissolve)

Supplementary Table S2 Summary of representative references on solar-driven desalination with the variation of salt concentration.

Material	Evaporation Rate	Solar-to-steam efficiency (%)	Salt deposition	Ref.
Janus fibrous	1.94	92.7	3.5wt.% 6 h	[1]
3D cone flowing evaporator with dust-free paper	3.22	142	3.5wt.% 8 h	[2]
PVA/SA hydrogel	2.21	90.7	3.5wt.% 63 days	[3]
Hydrophilic and hydrophobic double-layer structure	1.6	Unknown	3.5wt.% 60 h	[4]
Micro-evaporator (g-C_3N_4@PANI/PS)	1.3	90	3.5wt.% 15 h	[5]
Bilayer polypyrrole-sorghum straw	1.385	81.9	3.5wt.% 10 h	[6]
Construction of novel biomass-based solar evaporator	1.45	87.58	3.5wt.% 10 h	[7]
Modified lignin	2.259	Unknown	3.5wt.% 36 h	[8]
Ti_2O_3/polyurethane/polyacrylamide foam	2.15	140.42	3.5wt.% 2 h	[9]
Biochar-based interfacial evaporation	2.33	83.7	3.5wt.% 42 h	[10]
Bio-mimetic 3D structure	2.63	96	3.5wt.% 90 h	[11]
Aminated phenolated lignin	1.75	97.6	3.5wt.% 25 h	[12]
3D printing a biomimetic bridge-arch	1.64	91	10wt.% 200 h	[13]
Modified GO membrane	2.48	95.7	20wt.% 3 h	[14]
Hydrogel with a balsa wood sponge	2.13	86.7	20wt.% 30 days	[15]
PVA hydrogel evaporator	2.07	Unknown	20wt.% 24 h	[16]
Self-floating 3D SE with adiabatic foam particles	2.25	136.7	20wt.% 12 h	[17]
High-entropy-alloy-nanoparticles Enabled wood	2.58	Unknown	20wt.% 10 h	[18]
Channel-array/wood substrate	1.04	75	20wt.% 100 h	[19]
Carbon-fiber cloth	1.78	Unknown	25wt.% 7 h	[20]
3D-cone solar evaporator (Our work)	2.54	93.7	24.5wt.% 8 h	

Supplementary References

1. Dong, X.; Li, H.; Gao, L.; Chen, C.; Shi, X.; Du, Y.; Deng, H. Janus Fibrous Mats Based Suspended Type Evaporator for Salt Resistant Solar Desalination and Salt Recovery. *Small.* **2022**, *18*, 2107156.
2. Chen, Y.-Q.; Zhu, Y.-J.; Wang, Z.-Y.; Yu, H.-P.; Xiong, Z.-C. Salt-rejecting 3D cone flowing evaporator based on bilayer photothermal paper for high-performance solar seawater desalination. *J. Colloid Interface Sci.* **2024**, *660*, 370-380.
3. Li, F.; Li, N.; Wang, S.; Qiao, L.; Yu, L.; Murto, P.; Xu, X. Self-Repairing and Damage-Tolerant Hydrogels for Efficient Solar-Powered Water Purification and Desalination. *Adv. Funct. Mater.* **2021**, *31*, 2104464.
4. Yu, J.; Zhang, W.; Zhi, S.; Qi, X.; Chen, Y. Long Life and Salt Repellent Evaporator for Efficient Continuous Solar Desalination. *Sol. RRL.* **2023**, *7*, 2300347.
5. Xia, Q.; Wang, C.; Xu, N.; Yang, J.; Gao, G.; Ding, J. A Floating Integrated Solar Micro-Evaporator for Self-Cleaning Desalination and Organic Degradation. *Adv. Funct. Mater.* **2023**, *33*, 2214769.
6. Li, Y.; Xu, L.; Cai, J.; Liu, J.; Lv, B.; Chao, J.; Zhang, Q.; Zhao, Y. A Stable Bilayer Polypyrrole-Sorghum Straw Evaporator for Efficient Solar Steam Generation and Desalination. *Adv. Sustain. Syst.* **2022**, *6*, 2100342.
7. Liu, J.; Xu, L.; Li, Y.; Zhao, J.; Jia, X.; Chao, J.; Lv, B.; Zhao, Y. Construction of Novel Biomass-Based Solar Evaporator with Asymmetric Dual-Layer Structure for Water Desalination. *Adv. Sustain. Syst.* **2021**, *6*, 2100274.
8. Shao, Q.; Luo, Y.; Cao, M.; Qiu, X.; Zheng, D. Lignin with enhanced photothermal performance for the preparation of a sustainable solar-driven double-layer biomass evaporator. *Chem. Eng. J.* **2023**, *476*, 146678.
9. Li, L.; Zhao, P.; Wang, Z.; Hu, Z.; Wang, D.; Zhang, Y.; Li, S.; Li, C. Photothermal Ti2O3/polyurethane/polyacrylamide foam with high solar-evaporation efficiency. *Desalination.* **2023**, *567*, 117001.
10. Chen, S.; Sun, L.; Huang, Y.; Yang, D.; Zhou, M.; Zheng, D. Biochar-based interfacial evaporation materials derived from lignosulfonate for efficient desalination. *Carbon Neutraliz.* **2023**, *2*, 494-509.
11. Wu, L.; Dong, Z.; Cai, Z.; Ganapathy, T.; Fang, N.X.; Li, C.; Yu, C.; Zhang, Y.; Song, Y. Highly efficient three-dimensional solar evaporator for high salinity desalination by localized crystallization. *Nat. Commun.* **2020**, *11*, 521.
12. Yue, Y.; Wang, Y.; Bai, Y.; Han, J.; Cheng, W.; Han, G.; Wu, Q.; Jiang, J. A loofah-based all-day-round solar evaporator with phenolic lignin as the light-absorbing material for a highly efficient photothermal conversion. *Chem. Eng. J.* **2023**, *477*, 147298.
13. Zou, M.; Zhang, Y.; Cai, Z.; Li, C.; Sun, Z.; Yu, C.; Dong, Z.; Wu, L.; Song, Y. 3D Printing a Biomimetic Bridge-Arch Solar Evaporator for Eliminating Salt Accumulation with Desalination and Agricultural Applications. *Adv. Mater.* **2021**, *33*, e2102443.
14. Su, Y.; Liu, L.; Gao, X.; Yu, W.; Hong, Y.; Liu, C. A high-efficient and salt-rejecting 2D film for photothermal evaporation. *iScience.* **2023**, *26*, 107347.
15. Li, L.; He, N.; Yang, S.; Zhang, Q.; Zhang, H.; Wang, B.; Dong, T.; Wang, H.; Jiang, B.; Tang, D. Strong tough hydrogel solar evaporator with wood skeleton construction enabling ultra-durable brine desalination. *EcoMat.* **2022**, *5*, e12282.
16. Li, L.; He, N.; Jiang, B.; Yu, K.; Zhang, Q.; Zhang, H.; Tang, D.; Song, Y. Highly Salt-Resistant 3D Hydrogel Evaporator for Continuous Solar Desalination via Localized Crystallization. *Adv. Funct. Mater.* **2021**, *31*, 2104380.
17. Liu, H.; Chen, B.; Chen, Y.; Zhou, M.; Tian, F.; Li, Y.; Jiang, J.; Zhai, W. Bioinspired Self-Standing, Self-Floating 3D Solar Evaporators Breaking the Trade-Off between Salt Cycle and Heat Localization for Continuous Seawater Desalination. *Adv. Mater.* **2023**, *35*, e2301596.
18. Li, Y.; Ma, Y.; Liao, Y.; Ji, L.; Zhao, R.; Zhu, D.; Hu, X.; Qin, G.; Rong, H.; Zhang, X. High-Entropy-

Alloy-Nanoparticles Enabled Wood Evaporator for Efficient Photothermal Conversion and Sustainable Solar Desalination. *Adv. Energy Mater.* **2022**, *12*, 2203057.

19. Kuang, Y.; Chen, C.; He, S.; Hitz, E.M.; Wang, Y.; Gan, W.; Mi, R.; Hu, L. A High-Performance Self-Regenerating Solar Evaporator for Continuous Water Desalination. *Adv. Mater.* **2019**, *31*, e1900498.
20. Lam, D.V.; Nguyen, U.N.T.; Dung, D.T.; Kim, C.; Lim, M.; Kim, J.-H.; Lee, S.-M. Shape-transformable long-lasting superhydrophilic carbon cloth for sustainable solar vapor generation. *Chem. Eng. J.* **2024**, *481*, 148475.
